# Assessing the Long Term Impact of Phosphorus Fertilization on Phosphorus Loadings Using AnnAGNPS

**DOI:** 10.3390/ijerph8062181

**Published:** 2011-06-14

**Authors:** Yongping Yuan, Ronald L. Bingner, Martin A. Locke, Jim Stafford, Fred D. Theurer

**Affiliations:** 1 Environmental Sciences Division, Office of Research and Development, U.S. Environmental Protection Agency, 944 E. Harmon Ave., Las Vegas, NV 89119, USA; 2 Water Quality & Ecology Research Unit, National Sedimentation Laboratory, Agricultural Research Service, U.S. Department of Agriculture (USDA-ARS), 598 McElroy Dr., Oxford, MS 38655, USA; E-Mails: ron.bingner@ars.usda.gov (R.L.B.); martin.locke@ars.usda.gov (M.A.L.); 3 Natural Resources Conservation Service, U.S. Department of Agriculture (USDA-NRCS), Columbus, OH 43215, USA; E-Mail: jim.stafford@oh.usda.gov; 4 Water Quality & Quantity Team, Natural Resources Conservation Service, U.S. Department of Agriculture (USDA-NRCS), Beltsville, MD, USA; E-Mail: Fred.Theurer@verizon.net

**Keywords:** AnnAGNPS watershed modeling, phosphorus fertilization rates, phosphorus loss

## Abstract

High phosphorus (P) loss from agricultural fields has been an environmental concern because of potential water quality problems in streams and lakes. To better understand the process of P loss and evaluate the effects of different phosphorus fertilization rates on phosphorus losses, the USDA Annualized AGricultural Non-Point Source (AnnAGNPS) pollutant loading model was applied to the Ohio Upper Auglaize watershed, located in the southern portion of the Maumee River Basin. In this study, the AnnAGNPS model was calibrated using USGS monitored data; and then the effects of different phosphorus fertilization rates on phosphorus loadings were assessed. It was found that P loadings increase as fertilization rate increases, and long term higher P application would lead to much higher P loadings to the watershed outlet. The P loadings to the watershed outlet have a dramatic change after some time with higher P application rate. This dramatic change of P loading to the watershed outlet indicates that a “critical point” may exist in the soil at which soil P loss to water changes dramatically. Simulations with different initial soil P contents showed that the higher the initial soil P content is, the less time it takes to reach the “critical point” where P loadings to the watershed outlet increases dramatically. More research needs to be done to understand the processes involved in the transfer of P between the various stable, active and labile states in the soil to ensure that the model simulations are accurate. This finding may be useful in setting up future P application and management guidelines.

## 1. Introduction

The impact of high phosphorus (P) losses from watersheds into surface waters can lead to regional and national problems, ranging from the algal blooms and associated water quality problems in Lake Erie of the Great Lake systems in Northern Ohio [1], to large areas of hypoxia in the northern Gulf of Mexico [2–5]. Runoff resulting from agricultural practices has been identified as a primary source of P loads as they are transported into downstream waterbodies [6–9]. Odors and discoloration caused by decay of algae interfere with recreational and aesthetic water use, algae blooms shade submerged aquatic vegetation and reduce or eliminate photosynthesis and productivity, and algae may clog water treatment plant filters [10].

Phosphorus does not occur as abundantly as nitrogen (N) in soil. Total P in surface soils ranges from 0.005% to 0.15% [11]. Phosphorus is not as mobile as N, although it can be leached and lost through subsurface drainage [12]. Phosphorus is generally strongly adsorbed by soil. The sorption rate of P into the soil has been shown to be a dynamic factor [13] affected by percent clay and organic carbon, and P in solution [14]. The P adsorbed by sediment particles may be transported in overland flow. Phosphorus can also be dissolved as orthophosphate in the water and transported by surface and subsurface flow [12,15]. Surface runoff is the primary mechanism by which P is exported from most watersheds [16]. It is believed that among multiple sources of P, agricultural runoff from commercial fertilizer applications has the most significant impact on the algae blooms of Lake Erie [17]. In Northwest Ohio, where the algae blooms are the greatest, 60–80% of land use is agricultural. Scientists have proposed ways of reducing P loads to Lake Erie and other surface water systems. They include the reduction of P fertilization rates and adopted nutrient standards (NRCS 590); creation of filter strips (NRCS 393); Drainage Water Management (NRCS 554) and Structures for Water Control (NRCS 587); conservation Tillage; and cover crops (NRCS 340). Among all those recommended ways of reducing P loads to the Lake Erie, nutrient standards and/or fertilizer management seems a promising and economically sound way of reducing P load to Lake Erie. Understanding the impacts of long-term P fertilization on soil P content and P losses to surface runoff is critical for better fertilizer management. Given the expensive nature of long-term monitoring programs, computer models have been developed as an acceptable alternative for simulating the fate and transport of nutrients in agricultural soils, and for evaluating the effect of various agricultural management practices on nutrients losses to surface waters.

The overall objective of this study was to examine the long term effects of P fertilization on soil P content and runoff loss within the Upper Auglaize watershed in Ohio using the Annualized AGricultural Non Point Source Pollutant Loading model to improve the understanding of P losses so that farm management strategies might be sought to mitigate P losses.

## 2. Method and Procedures

### 2.1. AnnAGNPS Model Description

The Annualized AGricultural Non Point Source (AnnAGNPS) Pollutant Loading model is an advanced simulation model developed by the USDA-ARS and NRCS to help evaluate watershed responses to agricultural management practices [18]. It is a continuous simulation, daily time step, pollutant loading model designed to simulate water, sediment and chemical movement from agricultural watersheds [18].

The AnnAGNPS model evolved from the original single event AGNPS model [19], but includes significantly more advanced features than AGNPS. Because of the continuous nature of AnnAGNPS, daily climate information, which includes daily precipitation, maximum and minimum temperatures, dewpoint temperature, sky cover, and wind speed, is needed to account for temporal weather variations. The spatial variability of soils, land use, and topography within a watershed can be determined by dividing the watershed into many user-defined, homogeneous, drainage-area-determined cells. From individual cells, runoff, sediment and associated chemicals can be predicted from precipitation events that include rainfall, snowmelt and irrigation. AnnAGNPS simulates runoff, sediment, nutrients, and pesticides leaving the land surface and their transport through the channel system to the watershed outlet. Thus, the model has the capability to identify the sources of pollutants at their origin and to track those pollutants as they move through the watershed system. The complete AnnAGNPS model suite, which include programs, pre and post-processors, technical documentation, and user manuals, are currently available at http://www.ars.usda.gov/Research/docs.htm?docid=5199.

**Figure 1.**
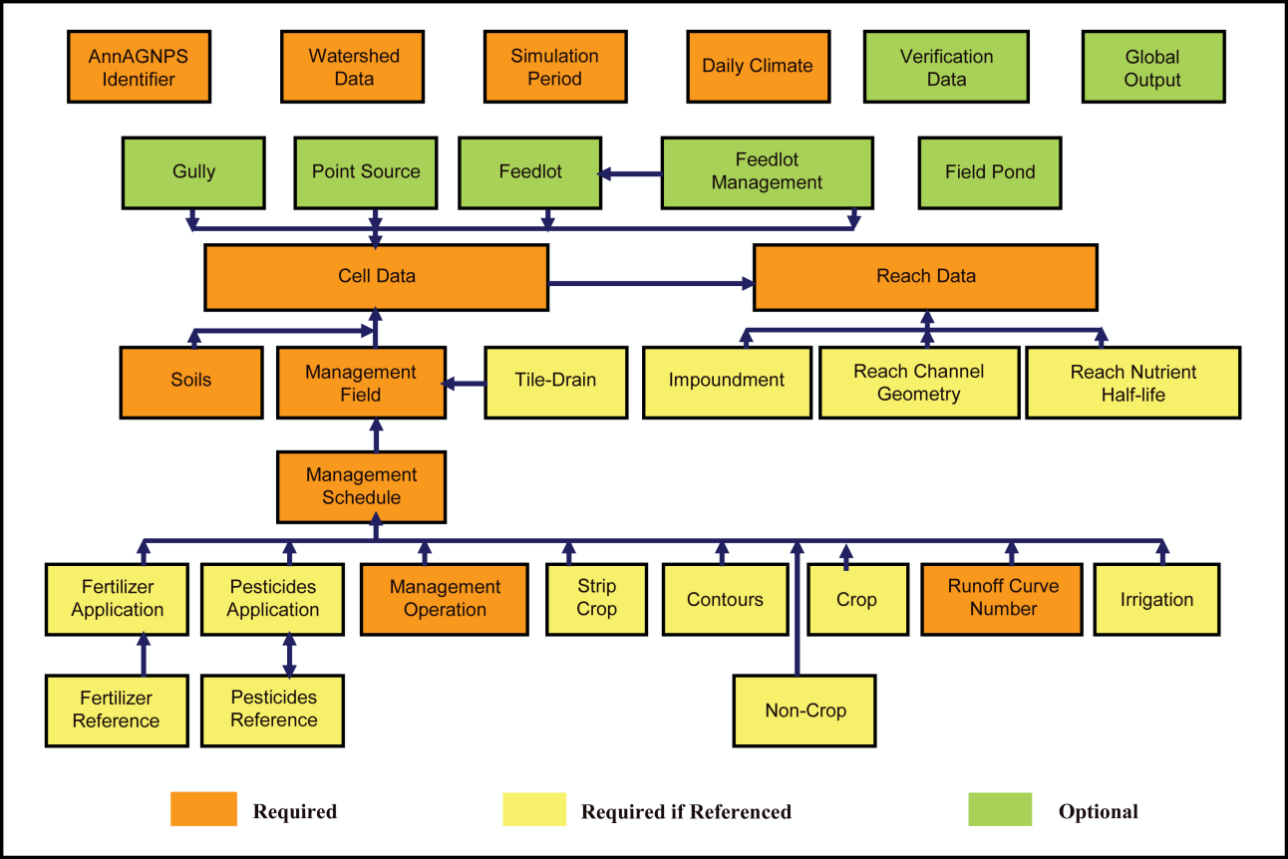
AnnAGNPS input data sections.

Input data sections utilized within the AnnAGNPS model are presented in Figure 1. Required input parameters include climate data, watershed physical information, and land management operations such as planting, fertilizer and pesticide applications, cultivation events, and harvesting. Daily climate information is required to account for temporal variation in weather and multiple climate files can be used to describe the spatial variability of weather. Output files can be generated to describe runoff, sediment and nutrient loadings on a daily, monthly, or yearly basis. Output information can be specified for any desired watershed source location such as specific cells, reaches, feedlots, or point sources.

### 2.2. The Upper Auglaize Watershed

**Figure 2.**
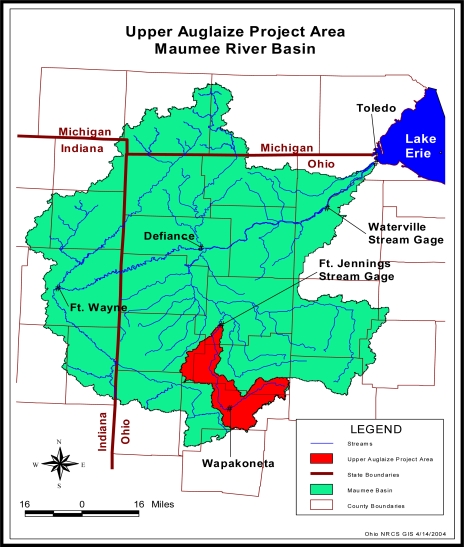
The Maumee River basin drainage network, Upper Auglaize watershed, and the Wapakoneta and Fort Jennings Gage Stations.

The Upper Auglaize (UA) watershed is located in portions of Auglaize, Allen, Putnam, and VanWert counties, Ohio, in the southern portion of the Maumee River Basin (Figure 2). The watershed encompasses 85,812 ha upstream of an outlet located at the Fort Jennings (04186500) U.S. Geological Survey (USGS) stream gage station (Figure 2). Land use is predominately agricultural, with 74% cropland, 11% grassland, 6% woodland, and 9% urban and other land uses. Corn and soybeans are the predominant crops grown in the watershed and together account for an estimated 83% of the agricultural cropland under cultivation and 62% of the total watershed area. Land-surface elevations in the UA watershed range from 233 to 361 m above sea level. Most soils in the UA watershed are nearly level to gently sloping; however, moraine areas and areas near streams can be steeper. In general, soils in the lower one-third of the watershed tend to be appreciably flatter than those in the upper two-thirds of the watershed. Blount (silt loam) and Pewamo (silty clay loam) are the major soil series in the watershed and together they are 62% of the watershed. These soils are characterized as somewhat poorly to very poorly drained with moderately slow permeability. Therefore, agricultural fields in the watershed are artificially drained to improve crop production. Subsurface drainage (tile drainage) systems have been installed to extend and improve drainage in areas serviced by an extensive network of drainage ditches. Common conservation practices applied in the watershed include grassed waterways, subsurface and surface drainage, conservation-tillage and no-tillage, grass filter strips, and erosion control structures.

### 2.3. Input Preparation of Existing Watershed Conditions

Using the Geographical Information System (GIS) digital data layers of elevation, soils, and land use, a majority of the data input requirements were developed by using a customized ArcView GIS interface [18]. Inputs developed from the ArcView GIS interface include physical information of the watershed and subwatershed (AnnAGNPS cell), such as boundary and size, land slope and slope direction, and channel reach descriptions. The ArcView GIS interface is also used to assign soil and land-use information to each cell by using the generated subwatershed and the soil and land-use GIS data layers. Additional steps to provide the model with the necessary inputs included developing the soil layer attributes to supplement the soil spatial layer, establishing the different crop operations and management data, and providing channel hydraulic characteristics. Those inputs can be organized using the AnnAGNPS Input Editor [18], a graphical user interface designed to aid users in selecting appropriate input parameters.

Soil information was obtained from the USDA-NRCS Soil Survey Geographic (SSURGO) Database [20]. SSURGO provides most of soil parameters required for an AnnAGNPS simulation, such as soil texture, erosive factor, hydraulic properties, pH value, and organic matter. Information on soil P was estimated based on soil test for P from the Comprehensive Nutrient Management Planning done by each county located in the watershed. Totally 298 samples were taken and analyzed for soil P test and the values range from 12–110 ppm with an average value of 36 ppm. Thus, soil total inorganic P was estimated as 36 ppm for the entire watershed. Geographical Information System (GIS) soil maps were used in conjunction with the subwatershed maps to determine the predominant soil assigned to each AnnAGNPS cell.

The characterization of the UA watershed land use, crop operation, and management during the simulation period was critical in generating estimates of the runoff, sediment and P loadings. AnnAGNPS has the capability of simulating watershed conditions with changing land use and crop management over long simulation periods. However, at the UA watershed scale, it was very difficult to characterize the long-term annual changes, including land use and field management practices, occurring in the watershed. Inputs for existing watershed conditions were established by using 1999–2002 LANDSAT imageries and a 4-year crop rotation derived from 1999–2002 field records [21]. A summary of the most prevalent crop rotations determined for the four-year land use data are shown in Table 1.

Rotation components are C (Corn), S (Soybeans), W (Wheat) and F (Fallow meaning permanent grass). The table combines four-year crop sequences that are equivalent except for the year in which they start. In other words, a rotation of CSCS is the same as SCSC for the sake of identifying existent crop rotations despite the fact that the sequences are offset by one year (the AnnAGNPS model keeps them separate by using an offset parameter). More details on development of land use and rotation sequences can be found in [21]. Because actual tillage information was not available for each field within the UA watershed, tillage type was applied on a random basis to each field such that the accumulative percent area of conventional, mulch, and no-till simulated for the 1999–2002 period was consistent with known percent areas for each tillage type for the same time period at the watershed scale. Percentages of tillage and land use for the UA watershed during 1999–2002 are summarized in Table 2.

AnnAGNPS allows for subsurface drainage systems to be simulated or not to be simulated for any given field during the model simulations. Since detailed information on subsurface drainage system location and drain diameter/spacing were not available, it was not possible to differentiate areas where subsurface drains were installed or the depth and spacing of any existing drainage system. Local experience substantiated that most fields in the watershed were subsurface drained to a very large extent. Therefore, the AnnAGNPS simulations were conducted with subsurface drainage conditions in all cells containing agricultural crops. A detailed methodology of subsurface drainage calculations are described in [22]. The option for entering subsurface drainage rate was used for subsurface drainage simulation. Model inputs of fertilizer application such as rates and extents were estimated based on interviews with four custom applicators operating in or near the UA watershed (Table 3). Fertilizer reference information was input based on AnnAGNPS guidelines and databases. Plant uptake was chosen through literature investigation [23] and listed in Table 4.

The runoff curve numbers were selected based on the National Engineering Handbook, Section 4 [24]. Crop characteristics and field management practices for various tillage operations were developed based on RUSLE [25] guidelines and local RUSLE databases. Climate data for AnnAGNPS simulation can be historically measured, synthetically generated using the climate generator program [26], or created through a combination of the two. A one-hundred-year synthetic weather dataset was developed and used for simulations in this study because historical weather data were not available. Complete information on weather generation can be found at the AnnAGNPS web site (http://www.ars.usda.gov/Research/docs.htm?docid=5199).

### 2.4. Model Calibration

Annual average (1979–2002) flow and sediment data collected at the Fort Jennings USGS stream gage station were used to calibrate AnnAGNPS simulated long-term annual average runoff and sediment loss. The long-term average annual data were chosen for calibration for the following reasons: (1) historical weather data were not available, and synthetic weather data were used for simulations (while synthetic weather data would not match historical weather data for an individual event, long-term synthetic weather statistics should reflect historical weather statistics); (2) land use, crop rotation, and management practices during the simulation period changed from year to year, and annual changes occurring in the watershed were not fully characterized by AnnAGNPS because of lack of information. The land use and management practices of 1999–2002 (Table 1 and Table 2) were considered to represent the existing situation of the watershed [21]. For simulations of existing watershed conditions, 100-year synthetic weather data were used, with the 4-year land use and tillage operation listed in Table 1 and Table 2 repeated for a 100-year period during simulations. However, the spatial distribution of actual tillage practices was not available for each crop field. From representative tillage transect data, the overall percentages of tillage types were known while the exact field-by-field values were not. Tillage type was applied on a random basis to each field to come up with the total amount of conventional, mulch, and no-till percentages reported for the counties in the watershed [21].

Land use and field management for the existing conditions were assumed to represent the calibration period of 1979–2002. Trial and error were performed to adjust AnnAGNPS parameters of drainage rate, curve numbers, amount of interception and management practices to produce the long-term average annual runoff and sediment loading close to that measured at the Fort Jennings USGS stream gage at the outlet. The range of adjustment of input parameters was limited to what was recommended in the references for this specific situation; thus, no specific calibration target was set up during calibration. Calibration was stopped when input values reached their specified limits. The maximum drainage rate was set to 12.5 mm/day (0.5 inches) based on local experience. The curve number for row crop which is the dominant land use in the watershed was selected from the Table 9 of the National Engineering Handbook-Section 4 [24]. The curve numbers used in model simulations after calibration are listed in Table 5. By default, AnnAGNPS assumes that interception is zero. A literature review suggests that interception ranges from 6–30% of the rainfall in crop land with residue cover [27] and the actual amount varies between 1.2 mm and 2.5 mm [27]. A value of 1.5 mm was used. For sediment, the only parameter adjusted was the gully delivery ratio and a value of 0.4 was used [28].

Following the calibration and simulation of existing conditions’ runoff and sediment loading, P loading from the watershed was simulated. No further calibration was performed for P loading because information on P loading was not available at the Fort Jennings USGS stream gage station. However, water quality data were available from the Maumee River at Waterville stream gage station (Figure 2). Water and pollutant loadings from the UA watershed go through the Waterville stream gage station before they enter the Lake Erie (Figure 2). Thus, AnnAGNPS simulated long-term average annual P loading was compared with average annual (1996–2003) P data collected at the Waterville stream gage station. Long-term average annual P loading was used for comparison for similar reasons discussed in runoff and sediment calibration.

### 2.5. Evaluation of the P Loadings from Different Fertilization Rates and Soil Initial P Contents

Following the modeling calibration, effects of P fertilizer rates and different initial soil P contents on P loadings were evaluated. The application rates of half of the existing application rate and one and half times of the existing application rate were analyzed. For initial soil P contents, four different levels as shown in Table 6 were analyzed. Level A reflects the existing P levels in the watershed. The existing P level in the watershed was determined using the soil P test data performed by each county in the watershed for the Comprehensive Nutrient Management Planning. Level B, C and D were determined as 4, 6 and 8 times of the level A respectively.

## 3. Results and Discussion

**Figure 3.**
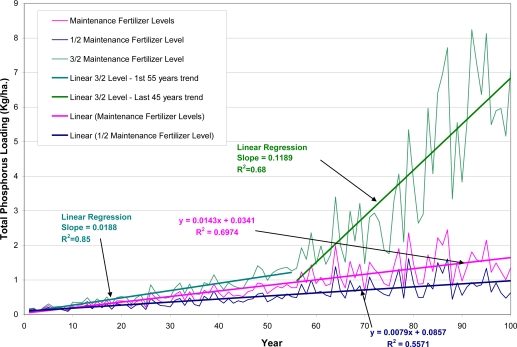
Annual phosphorus loading at the Upper Auglaize Watershed Outlet from different P application rates.

**Figure 4.**
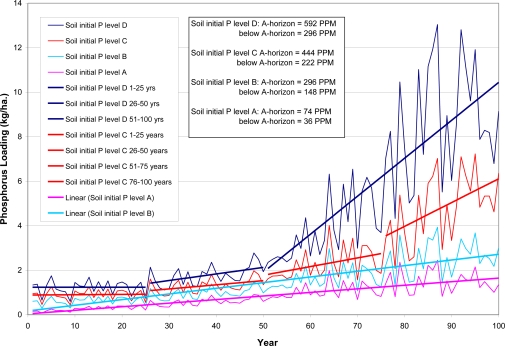
Annual phosphorus loading at the Upper Auglaize Watershed Outlet from Different Initial Soil P levels for the existing P application rate.

Model calibration results are presented in Table 7. Results of P loadings from different P application rates are shown in Figure 3; and results of P loadings from different initial P contents are displayed in Figure 4.

### 3.1. Model Calibration

Annual average runoff (1979–2002) observed at the Fort Jennings USGS stream gage station was 254 mm. After calibration, the simulated 100-year annual average runoff was 254 mm, which consisted of 163.6 mm from direct surface runoff and 90.4 mm from subsurface quick return flow (Table 7). Subsurface drainage flow was the major component of subsurface quick return flow. Annual average sediment loading (1979–2002) observed at the Fort Jennings USGS stream gage station was 0.753 T/ha/yr. After calibration, the simulated 100-year annual average sediment loading was 0.771 T/ha/yr (Table 7). More details on runoff and sediment calibration and their changes from different management scenarios can be found in [21]. Runoff and sediment calibration is important for this study because parameters used during calibration are the basis for P loading and additional alternative scenarios evaluation.

Evaluating and calibrating the model in a more intensive way, such as comparison of annual runoff and sediment, was not possible because historical weather data were not available for the study site [21]. In addition, when and where land use changed and how field management operation (including planting, harvesting, and tillage operations) changed during 1979–2002 were not known. The 4-year land use and management practices of 1999–2002 (Table 1 and Table 2) were assumed to represent the condition for 1979–2002 calibration period, and they were repeated during the simulation period. Therefore, the calibration of the model is limited to average annual. The average annual reflects the long-term trend that occurred in the watershed over the years; thus, the critical parameters impacting runoff and sediment loadings from the watershed can still be calibrated to better reflect the actual conditions of the watershed.

The simulated 100-year average annual agricultural total P was 0.85 kg/ha/yr (Table 7) using those calibrated parameters for runoff and sediment. Average annual total P loading (1996–2003) observed at the Waterville stream gage station was 1.09 kg/ha/yr, which included point source and nonpoint source P loadings. No addition calibration was performed because it is very difficult to separate agricultural nonpoint source P loading from total P loading which includes point source and nonpoint source at the Waterville stream gage station. In addition, the sensitive parameters for P loading such as P fertilizer application rate, soil P concentration and plant uptake [23] were carefully chosen to best represent the watershed condition. Further adjusting those parameters may result in loss of accuracy in representing the watershed condition. For instance, fertilizer application rates were directly obtained from farmer surveys and soil P concentration was estimated based on P soils test data from the Comprehensive Nutrient Management Planning done by each county located in the watershed. Finally, to evaluate the effects of different levels of P fertilizer application on P loading, the relative impact of those different levels of P application on P loading is needed. The comparison of their relative impacts could help researchers better understand the P transport processes which can be used for future nutrient management and decision making.

### 3.2. Analysis of Annual P Loadings from Different P Application Rates

The average annual total P was 0.54 kg/ha/yr for half of the existing P application rate, 0.85 kg/ha/yr for existing P application rate, and 2.12 kg/ha/yr for one and half times of the existing application rate. Although the application rate is increased by 100% from half of existing application rate to existing application rate, the total P loading is increased by 57%. However, the total P loading increased by 150% from existing application rate to one and half times of the existing application rate which is only by 50% increase in fertilizer application. This indicated that at certain levels, the P loadings increased dramatically. Research has shown that the P losses from agricultural fields increased as soil P content increased [10], and when the soil P content exceeds soil P adsorption capacity, the P losses from agricultural fields increased dramatically [29].

AnnAGNPS simulation results of annual loading over 100-year indicate that P loading has an increasing trend, and this increasing tread increased significantly for the one and half of the existing application rate (Figure 3). This increasing trend in P loading can not be accounted for by the variability in runoff because runoff does not have a significant increasing trend, nor do sediment loadings (Figures not shown). When P fertilizer was increased to one and half times of the existing application rate, there is a significant change (jump) in the trend near approximately the 45th year of simulation (Figure 3). However, this abrupt change in loading does not appear for the existing application rate and one half of the existing application rate (Figure 3). More research is needed to study this change in order to improve the model simulations such as varying approaches to account for the sorption of P that would better describe the dynamic effect of fertilizer applications on labile P [14]. The increases in loadings are much more modest (Figure 3) for the existing application rate and 0.5 of the existing application rate. This suggested that under sustained, long term high P fertilizer applications, there is a buildup in soil P level that results in significant higher P loadings at the watershed outlet. Field studies done by others also show that P application to agricultural soils exceeding P uptake by crops leads to P accumulation in soils in the long term, and this accumulation results in higher P loss to water [10,30,31]. Studies also show that it could take up to 10–50 years to see this build up depending on the soil properties, amount of P applied and cropping systems [30]. The dramatic change (this change point is referred as “critical point” in the rest of the paper) in P loadings after 45 years for the one and half times of the existing application rate explains the phenomena of much higher average annual P loading (2.12 kg/ha/yr) of this application rate.

### 3.3. Analysis of Annual P Loadings from Different Soil Initial P Contents

AnnAGNPS simulation results of annual loadings over 100-year using the existing application rate, but with different initial levels of soil P content indicate that if higher initial levels of soil P exist, even with the existing levels of P fertilizer application rate can produce the significant increase (jump) in P loadings over time (Figure 4). The higher the initial soil P content is, the bigger and the sooner the abrupt change occurs. As shown in Figure 4, it takes about 75 years to see the jump in P loading for the initial soil level C (Table 6), and it takes about 50 years to see the jump in P loading for the initial soil level D (Table 6). Because the higher level of initial P, it takes less time to build up to the “critical point” to change the soil P loss to water.

### 3.4. Analysis of Soil in Situ P for Various AnnAGNPS Cells

A different but parallel way to look at what is happening in simulated P fertilizer applications is to examine the changes of phosphorus levels in the soil over time. The AnnAGNPS model provides results on the *in situ* soil P changes over time during the model simulation for each subwatershed (AnnAGNPS cell or computational area).

**Figure 5.**
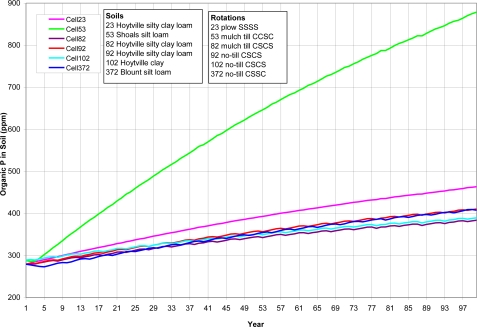
*In situ* organic soil P for top 200-mm of soil for six cells. Cells were arbitrarily chosen to represent different soils, crop rotations and tillage. SSSS refers to continuous soybean during 1999–2002, CCSC refers to corn-corn-soybean-corn rotation during 1999–2002, CSCS refers to corn-soybean-corn-soybean rotation during 1999–2002, and CSSC refers to corn- soybean -soybean-corn rotation during 1999–2002.

**Figure 6.**
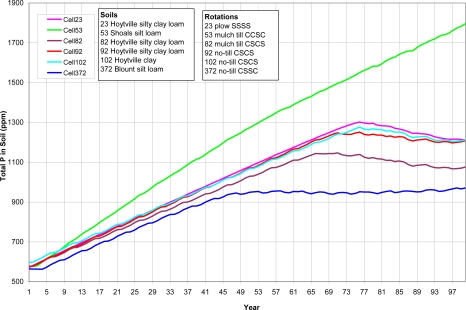
*In situ* total soil P for top 200-mm of the soil for six cells. Cells were arbitrarily chosen to represent different soils, crop rotations and tillage. SSSS refers to continuous soybean during 1999–2002, CCSC refers to corn-corn-soybean-corn rotation during 1999–2002, CSCS refers to corn-soybean-corn-soybean rotation during 1999–2002, and CSSC refers to corn- soybean -soybean-corn rotation during 1999–2002.

**Figure 7.**
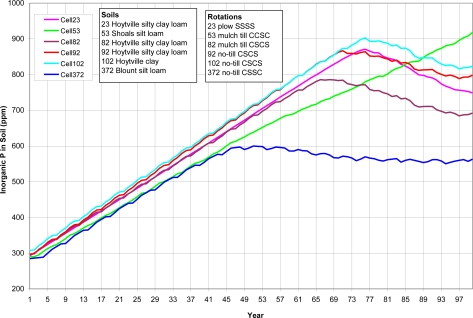
*In situ* inorganic soil P for top 200-mm of the soil for six cells. Cells were arbitrarily chosen to represent different soils, crop rotations and tillage. SSSS refers to continuous soybean during 1999–2002, CCSC refers to corn-corn-soybean-corn rotation during 1999–2002, CSCS refers to corn-soybean-corn-soybean rotation during 1999–2002, and CSSC refers to corn- soybean -soybean-corn rotation during 1999–2002.

**Figure 8.**
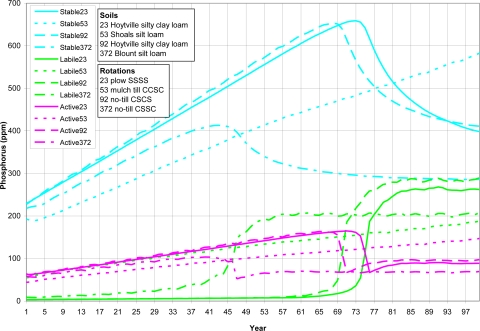
*In situ* stable, active and labile (inorganic) soil P top 200-mm of soil for four cells.

A variety of watershed cells were chosen to represent different soils, crop rotations and tillage (Table 8). As shown from Figure 5 to Figure 8 (existing P application rate with soil initial P level D), the AnnAGNPS model tracks total soil P, organic P and inorganic soil P from each computational area. The inorganic P is further broken down into labile P (P readily available for plant uptake), active P (P that is more or less reversibly adsorbed to the soil), and stable P (adsorbed P that is “fixed” as discrete insoluble P minerals or relatively irreversibly chemisorbed to the soil adsorption complex). Soil organic P content is increasing over the entire simulation period (Figure 5). Soil total P is increasing over the entire simulation period for cell 53 with shoals silt loam, a different soil type than the others; while the soil total P is increasing until it reaches the “critical point” for other five cells, then soil total P is decreasing slightly except for cell 372 (Blount silt loam) in which the soil total P stays constant (Figure 6). The inorganic P presents the similar trend as total P except that the inorganic P decreases for all five cells after the “critical point” (Figure 7). Due to different soil properties of cells, this value of the “critical point” is different for different cells. As the total phosphorus builds up in the soil, a sudden increase in the labile portion occurs (Figure 8). This result suggests that when there is a buildup of P in the soil that at some point the amount subject to loss also increases dramatically (Figure 8). This seems to confirm the findings of others that “a change point” occurs where for greater soil P concentrations, significantly greater P loss occurs in both surface and subsurface runoff [6].

Again, the timing to this “critical point” is different for cells depending primarily on the soil properties and other factors such as crop rotations and tillage. The cell 372 (Blount silt loam) with no-till corn-soybean-soybean-corn rotation reaches the change point at about 45 years, while cell 92 (Hoytville silty clay loam) with corn-soybean-corn-soybean rotation reaches the change point at about 70 years (Figure 8). The timing as the large increase in P loadings as seen in Figure 3 and Figure 4 probably reflects the sudden increase in the labile P. Since P loadings as seen in Figure 3 and Figure 4 reflect the overall watershed response at the outlet, the timing to sudden increase in loadings would not match the exact timing of individual cells of sudden increase in labile P.

These results may be useful in setting up future P fertilizer application and management guidelines at the watershed scale. Although findings from this study are consistent with field observations at other locations, but the actual value of “critical points” and P loadings obtained from this study may not be comparable with results obtained from other locations because P losses are very complicated processes and it is impacted by many different factors [32]. Future watershed modeling work would focus on targeting critical areas for P management practices implementation to achieve maximum water quality benefits.

## 4. Summary and Conclusions

AnnAGNPS model was applied to the Ohio UA watershed to evaluate the impact of P fertilization rates on P loadings and soil P content changes. The model was calibrated using average annual data collected at the Fort Jennings USGS gauging station because historical weather data were not available, and 100-year synthetic weather data was used for simulation. Although significant efforts were spent in characterizing land use, tillage, crop rotation, and management practices during model calibration, the day by day temporal and field by field spatial variations of the information were not fully represented in the model. The synthetic weather data would not match historical weather data for an individual event, long-term synthetic weather statistics should reflect historical weather statistics; furthermore, the average annual reflects the long-term trend that occurred in the watershed over the years; thus, the critical parameters impacting runoff and sediment loadings from the watershed can still be calibrated to better reflect the actual conditions of the watershed.

AnnAGNPS simulation results of different P fertilization rates showed that P loadings increase as fertilization rate increases, and long term builds up of soil P would lead to much higher loadings of P to the watershed outlet. This dramatic change of P loading to the watershed outlet indicates that a “critical point” may exist in the soil at which soil P loss to water changes dramatically. The higher the initial soil P content is, the less time it takes to reach the “critical point”. Analysis of soil P changes showed that as the total soil P builds up in the soil, a sudden increase in the labile portion occurs, which results in dramatic increase in P loadings to surface runoff. This finding seems to confirm the findings of others and may be useful in setting up P application and management guidelines, however, more research needs to be done to understand the processes involved in the transfer of P between the various stable, active and labile states in the soil to ensure that the model simulations are accurate.

## Figures and Tables

**Table 1.** Crop rotations summarized for the 4-year land use, C (Corn), S (Soybeans), W (Wheat) and F (Fallow meaning permanent grass).

**Rotation**	**Area (ha)**	**Percent of agricultural land use**	**Accumulated percent**
CSCS	16,894	21.9%	21.9%
CCCS	10,833	14.1%	36.0%
CSSS	6,286	8.2%	44.1%
CCSS	5,741	7.5%	51.6%
CCSW	5,680	7.4%	59.0%
CSWS	4,016	5.2%	64.2%
CSCW	3,407	4.4%	68.6%
CSSW	3,389	4.4%	73.0%
CCFF	1,391	1.8%	74.8%
CWSW	1,387	1.8%	76.6%
CWSS	1,295	1.7%	78.3%
SSSS	1,184	1.5%	79.8%
CSWW	1,182	1.5%	81.3%
CCCW	1,171	1.5%	82.9%
CCWS	1,121	1.5%	84.3%
CCCC	1,121	1.5%	85.8%
SSSW	1,104	1.4%	87.2%
FFWC	1,057	1.4%	88.6%
CCSF	575	0.7%	89.3%
CWFW	559	0.7%	90.1%
FFFW	431	0.6%	90.6%

**Table 2.** Upper Auglaize watershed 4-year crop, tillage, and land-use distribution in percent, the total area is 85,812 hectares.

**Landuse**	**Tillage**	**1999**	**2000**	**2001**	**2002**
Corn	Conventional	10.1%	13.1%	10.5%	10.5%
	Mulch till	18.7%	17.0%	20.3%	17.9%
	No till	10.4%	14.1%	12.2%	14.0%
	Total	39.3%	44.2%	43.0%	42.3%
	
Beans	Conventional	8.7%	6.0%	7.4%	9.4%
	Mulch till	9.6%	16.8%	11.5%	13.7%
	No till	11.8%	11.1%	13.7%	11.2%
	Total	30.0%	33.9%	32.5%	34.2%
	
Wheat	Conventional	1.9%	2.6%	3.7%	1.6%
	Mulch till	5.3%	3.8%	4.3%	2.7%
	No till	5.2%	4.6%	3.1%	3.8%
	Total	12.4%	10.9%	11.1%	8.0%
	
Grass	Conventional	1.4%	0.4%	0.5%	0.6%
	Mulch till	4.2%	0.2%	1.7%	3.7%
	No till	2.7%	0.4%	1.1%	1.2%
	Continuous	0.4%	0.4%	0.4%	0.4%
	Total	8.7%	1.4%	3.7%	5.8%
	
Forest		5.6%	5.6%	5.6%	5.6%
	Residential	2.0%	2.0%	2.0%	2.0%
	Roads	1.4%	1.4%	1.4%	1.4%
	Commercial	0.5%	0.5%	0.5%	0.5%
	Water	0.1%	0.1%	0.1%	0.1%
	Grand Total	100.0%	100.0%	100.0%	100.0%

**Table 3.** Fertilizer application for main crops.

**Crop Type**	**Nitrogen (kg/ha.)**	**P_2_O_5_(kg/ha.)**
Corn	157	50
Soybean	0	34
Wheat	65	45
Alfalfa	0	73

**Table 4.** Plant P uptake ratio.

**Corn**	**Soybean**	**Wheat**
0.0026	0.0095	0.0025

**Table 5.** Curve numbers used for model simulations after calibration.

**AnnAGNPS land cover**	**Land cover class from Table 9 of the NHD-4 (SCS, 1985)**	**Curve Number**			
		**Hydrological soil group**			
		**A**	**B**	**C**	**D**
Row crop with NT*	Row crop contoured and terraced (good)	62	71	78	81
Row crop with RT*	Row crop contoured with crop residue (good)	64	74	81	85
Row crop with CT*	Row crop straight row (poor)	72	81	88	91
Small grain with NT*	Small grain contoured and terraced (good)	59	70	78	81
Small grain with RT*	Small grain contoured and terraced (good)	60	72	80	84
Small grain with CT*	Small grain contoured and terraced (good)	64	75	83	86
Fallow	Fallow with crop residue (good)	74	83	88	90
Forest	Woods (good)	30	55	70	77
Commercial	Residential (38% impervious)	61	75	83	87
Residential	Residential (38% impervious)	61	75	83	87
Roads	Roads (paved w/ditch)	83	89	92	93

* NT refers to no-tillage, RT refers to reduced tillage and CT refers to conventional tillage.

**Table 6.** Various initial soil total inorganic P contents used for AnnAGNPS simulations.

**Initial Soil Total Inorganic P Content (mg/kg or PPM)**	**Top Soil Layer**	**Bottom Soil Layer**
A*	74	36
B	296	148
C	444	222
D	592	296

* Level A was determined based on the soil P test data from each county in the watershed for the Comprehensive Nutrient Management Planning.

**Table 7.** Post-calibration model outputs of runoff, sediment and phosphorous as compared to observed values for existing watershed conditions.

**Item**	**AnnAGNPS Simulation**	**USGS Observation**
Watershed annual average direct surface runoff (mm)	162.6	
Watershed annual average subsurface flow (mm)	91.4	
Watershed annual average total runoff (mm)	254.0	254.0
Sediment loading at the watershed outlet (t/ha/Yr)	0.771	0.753
Total P loading at the Waterville gage (kg/ha/Yr)	0.85	1.09

**Table 8.** List of cells which *in situ* P were analyzed during simulation period and their soil type and land use during 1999–2002.

**Cell ID**	**Soils**	**Field Management**
23	Hoytville silty clay loam	Conventional-till continuous soybean (SSSS)
53	Shoals silt loam	Reduced-till corn-corn-soybean-corn (CCSC)
82	Hoytville silty clay loam	Reduced-till corn-soybean-corn-soybean (CSCS)
92	Hoytville silty clay loam	No-till corn-soybean-corn-soybean (CSCS)
102	Hoytville clay	No-till corn-soybean-corn-soybean (CSCS)
372	Blount silt loam	No-till corn-soybean-soybean-corn (CSSC)
